# Using Rose Bengal photosensitizer for controlling *Aphis gossypii* and reducing transmission of zucchini yellow mosaic virus on squash plants

**DOI:** 10.1038/s41598-026-51365-6

**Published:** 2026-05-19

**Authors:** Doaa Z. Soliman, Mohamed S. El-Masarawy, Ahmed A. Kheder, Alexandra A. El-Helaly, Abeer S. Abd El-Wahab, Sameha A. Metwally

**Affiliations:** 1 Department of Plant Pathology, Faculty of Agriculture, Cairo University, Giza, Egypt; 2 Department of Economic Entomology and Pesticides, Faculty of Agriculture, Cairo University, Giza, Egypt; 3 Virus and Phytoplasma Department, Plant Pathology Research Institute, Agricultural Research Center, Giza, Egypt

**Keywords:** Rose Bengal, Photosensitizer, *Aphis gossypii*, ZYMV, *Cucurbita pepo*, Aphid transmission, Biotechnology, Microbiology, Plant sciences

## Abstract

The cotton aphid, *Aphis gossypii*, exhibits the highest transmission rate of zucchini yellow mosaic virus (ZYMV). This study aimed to evaluate the lethal effect of Rose Bengal photosensitizer (RBPS) on aphids and transmission of ZYMV on squash plants. Five concentrations of 0.1, 1, 10, 100, and 1000 ppm were used on adults of a virus-free aphid. Results confirmed that the highest mortality (100%) occurred after 72h of treatment with a concentration of 1000 ppm, where LC_50_ was 1.66 and 0.0807 ppm after 24h and 72h, respectively under sunlight exposure. The reverse transcription polymerase chain reaction (RT-PCR) of the virus isolate confirmed the presence of ZYMV. A sequence analysis was carried out and given a GenBank accession number PZ289840.1. Transmission studies showed that after pre-acquisition treatment by RBPS, the ability of aphids to pick up the virus was reduced to 23.3% with a 74.3% transmission reduction. In the pre-inoculation treatment, RBPS reduced the efficiency of *A. gossypii* in the virus transmission to 40.0% with a reduction of 55.6%. Using RBPS before both virus acquisition and inoculation gave the lowest transmission rate (13.3%) and highest reduction (86.7%). This method will be promising for controlling virus spread by aphids under the field conditions.

**Supplementary Information:**

The online version contains supplementary material available at 10.1038/s41598-026-51365-6.

## Introduction

The cotton aphid, *Aphis gossypii* (Homoptera: Aphididae) is a polyphagous insect and a major pest of various plants. Among cucurbit vegetables, it can seriously affect watermelons, cucumbers, cantaloupes, squash, and pumpkins. It absorbs plant sap. Honeydew is excreted by the aphids, allowing sooty molds to grow, resulting in a decrease in the quantity and quality of the produce. The aphids inoculate toxins and serve as vectors for viruses to several plants^1^.

Zucchini (*Cucurbita pepo L.*) is one of the most important summer crops belonging to the Cucurbitaceae family^2^. Its importance extends beyond its role as a food source; it also boasts medicinal benefits. This nutrient-rich and versatile vegetable possesses significant and scientifically recognized medicinal properties, including antioxidant, antimicrobial, and anti-inflammatory effects. Due to its vitamin and mineral content, various parts of this plant are traditionally used to treat inflammation, constipation, urinary tract problems, prostate enlargement, and skin conditions^3^. Optimal water and nutrient management can achieve total zucchini yields of approximately 40–54 tons per hectare by 2025^4^.

Zucchini yellow mosaic virus (ZYMV) is a member of the genus *Potyvirus* within the family *Potyviridae*. As other members of the genus *potyvirus*, it possesses flexuous, filamentous particles approximately 750 nm in length^5^. Its genome consists of a single-stranded RNA (9,600 nucleotides)^6^.ZYMV is primarily transmitted by aphid vectors and can also be transmitted through infected seeds^7,8^. The virus can be transmitted by at least 26 aphid species, as well as through mechanical means^9,10^ Field studies have identified *A. gossypii*, *Myzus persicae*, and *A. craccivora* as the primary aphid species responsible for ZYMV transmission^11^ Transmission efficiency varies among species: *A. gossypii* exhibits the highest transmission rate, followed by *M. persicae* and then *A. craccivora*^12,13^ *A. gossypii* and *M. persicae* are known to transmit the virus non-persistently to crops such as zucchini and melons^14–20^. Additionally, *A. gossypii* populations were observed to be more abundant on infected squash plants compared to healthy ones^14,17^. The aphid vectors spread the virus not only within cucurbit-cultivated fields but also within grown weeds. Weeds play, indeed, a critical role in spreading the virus, whereas these act as natural reservoirs of the virus during the offseason, and serve as alternate hosts of pathogens spreading the infection at the inception of the growing season^21–23^.

The ZYMV was identified in Italy in 1973, as reported by Lisa et al^5^.. Later, it was identified throughout the world. In Egypt, it was first discovered in 1983, by Provvidenti et al.^24^. Globally, ZYMV is one of the most economically important viruses of cucurbit crops^25^. Mosaic, leaf malformation, blistering, and plant dwarfing are typical symptoms of infected cucurbit plants. Infected fruits are deformed and discolored, making them unmarketable. Early infection results in up to 95% losses in fruit sales^26–28^.

Noting that plant viruses have no cure, evading infection is, then, highly sought^29,30^. Some of the most effective control strategies for ZYMV include cultivating resistant varieties, controlling virus vectors, and destroying alternate hosts of pathogens^31^. This specifically applies to ZYMV, being widely reported as extremely harmful in highly automated production regions as well as traditional agro-ecosystems^25^. The continuing traditional control of *A. gossypii* by using synthetic insecticides causes disruption of natural enemies, create toxic residues in the crops, and induce resistance development and undesirable impacts on non-target organisms^32^. Therefore, new treatments and compounds with less harmful environmental impacts had to be identified to combat active pests^33^.

Photosensitizer (PS) with a light beam, is not toxic in the dark, but induces the production of highly reactive oxygen species (ROS), such as singlet oxygen (^1^O_2_), upon light illumination. Upon light illumination, the PS is activated into a singlet excited state and then into a triplet state following intersystem crossing. In its triplet state, the PS undergoes electron or proton transfer to produce superoxide anion and hydroxyl radical, or transfers its energy into oxygen to produce O_2_^34^. Rose Bengal (4, 5, 6, 7-tetrachloro-20, 40, 50, 70-tetraiodofluorescein disodium, RB) is one of the photosensitizers, which is a dye belonging to the fluorescein family^35^. This amphiphilic chemical molecule has turned as a promising approach for controlling insects recently.

The main objective of this study is to evaluate the toxicity effect of Rose Bengal photosensitizer (RBPS) on cotton aphid vector, *A. gossypii,* transmission of Zucchini yellow mosaic virus (ZYMV) on squash (*Cucurbita pepo* L.) plants under semi-field conditions.

## Materials and methods

### Aphid collection and identification

Aphids were collected from the field and maintained in insect-proof cages under controlled laboratory conditions at a temperature of 25 ± 2 °C, relative humidity of 60–70%, and a 16:8 h (light:dark) photoperiod. Specimens were then identified to the species level using the morphological identification key developed by Blackman & Eastop^36^, Species identification was performed based on diagnostic morphological characters, including body shape, coloration, siphunculi structure, cauda shape, and antennal segmentation. According to these characteristics, the aphid specimens were identified as *A. gossypii* Glover.

### Maintenance of virus-free aphid culture

A virus-free culture of *A. gossypii* was established and maintained for experimental use. Cotton seedlings were used as host plants for rearing the aphids. Field-collected apterous adult females were individually placed on moistened filter paper discs (Whatman No. 1, 9 cm diameter, pore size ~ 11 µm) inside sterile Petri dishes until reproduction began to reproduce (viviparity). The first-generation nymphs were carefully transferred using a camel’s hairbrush (size zero) onto healthy cotton plants grown in pots. Each pot was enclosed in a cylindrical glass cage covered with muslin cloth to prevent contamination.

**Fig. 1:**
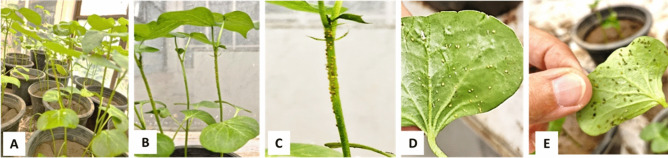
Culture of *A. gossypii* virus free reared on cotton seedling.

The aphid cultures were maintained under glasshouse conditions in a greenhouse belonging to the Plant Pathology Department, Faculty of Agriculture, Cairo University, Giza, Egypt (Fig. 1), with supplemental lighting from fluorescent lamps during short photoperiods. To ensure that the aphid colony was free of viruses, a subset of aphids was allowed to feed for 24 h on healthy indicator plants. These plants were then monitored for symptom development. If no symptoms appeared, the aphids were, then, confirmed to be virus-free^37^. For farther confirmation the non-viruliferous aphid were tested by PCR (Fig. 9).

### Bioassay

**Fig. 2:**
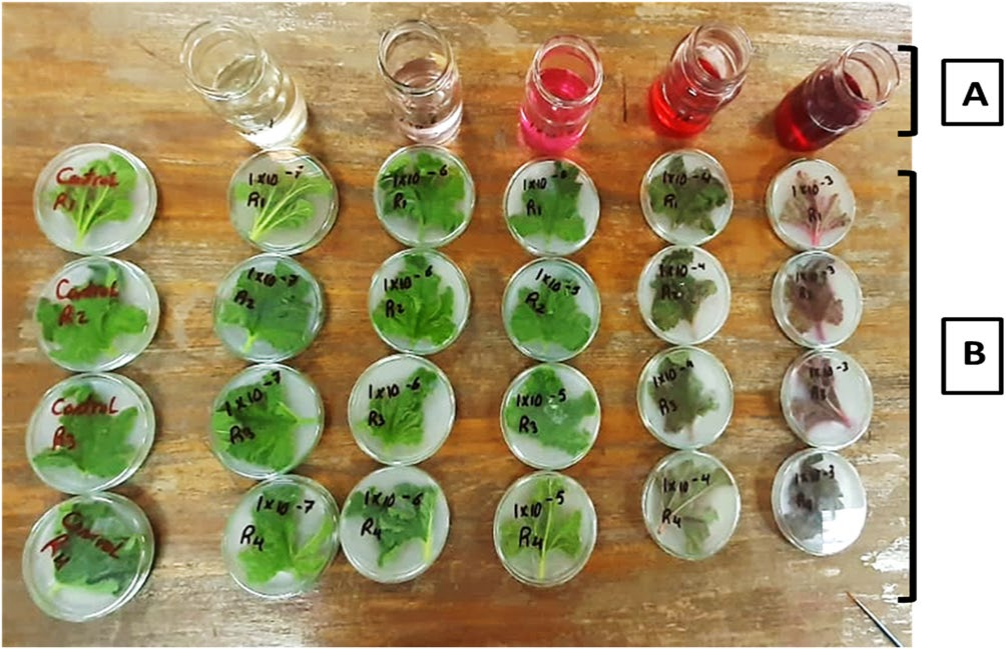
Lethal effect assessment of Rose Bengal (RBPS) on *A. gossypii*. (**A**) Five concentrations of (RBPS), (**B**) Treated squash leaves with adults of *A. gossypii* placed.

Five concentrations of Rose Bengal photosensitizer (RBPS) (0.1, 1, 10, 100, and 1000 ppm) were used against the adult of *A. gossypii* by the leaf dipping technique of squash plants. Four replicates were devoted for each tested concentration with 20 individuals for each. Fresh leaves of squash plants were dipped into each of the different tested concentrations for 5 min. The leaves were placed on paper towels until dried, then transformed into petri dish plates of 5 cm diameter poured with 5% agar concentration to maintain freshness over extended time (Fig. 2). The adults were gently placed by fine camel-hairbrush to feed into the treated leaves^38^. Petri dishes were closed and kept in dark under the laboratory conditions of 27–28°C and 75–80% R.H. according to El-Bendary, & El-Helaly^39^. Control treatments received the same protocol using distilled water. The experiment was checked after 24 and 72 h from exposure and recording numbers of dead. The mortality percentages were corrected according to Abbott’s formula^40^ and the LC_50_ and LC_90_ values for all treatments were calculated after 24 and 72 h of treatment.

### Source of infected plants with ZYMV

Samples of squash (*Cucurbita pepo* L.) plants were collected from Dakahlia Governorate, Egypt, showing mosaic, blisters, and leaf deformation, typical ZYMV-like symptoms. Samples were preserved with anhydrous calcium chloride until they were processed for the detection of viral isolates and experiments related to the subject field.

### Mechanical inoculation and maintenance of virus isolate

The isolate under study was maintained on squash plants cv. Eskandrani by serial mechanical transmissions. For mechanical inoculation, infected samples were ground (1:2w/v) in 0.1 M phosphate buffer saline, pH 7.4. The infectious sap was mixed with celite before being inoculated into healthy squash plants. The inoculated leaves were washed thoroughly with water. Inoculated plants were kept in the insect-proofed glasshouse in a greenhouse belonging to the Plant Pathology Department, Faculty of Agriculture, Cairo University, Giza, Egypt.

## Molecular detection of ZYMV isolate

### RNA extraction

Four samples were collected from infected squash plants cv. Eskandrani growing in the insect-proofed glasshouse displaying different virus-like symptoms after several series of viral mechanical inoculations. Total RNA was extracted from the four samples in addition to an extract from healthy plants. 100 mg of each sample were ground using a Total RNA Mini Kit (plant 2/2) Geneaid (Cat. NO. RP300, Taiwan), considering the manufacturer’s instructions.

### Reverse transcription and polymerase chain reaction (RT-PCR)

One-step RT-PCR was applied to the total RNA using the Verso 1-Step RT-PCR ReddyMix Kit (Thermo Fisher Scientific, Cat. No. AB1454LDB, USA). The following cycling parameters were applied: reverse transcription at 42 °C for 30 min, initial denaturation at 94 °C for 2 min, and then 35 cycles of denaturation for 30 s at 94 °C, annealing for 30 s at 65 °C, and extension for 1 min at 72 °C, followed by a final elongation step at 72 °C for 10 min. RT-PCR was conducted using a pair of ZYMV-specific primers; forward primer (ZY-2: 5’- GCTCCATACATAGCTGAGACAGC-3’), derived from the nuclear inclusion protein b (NIb) gene and reverse primer (ZY-3: 5’-TAGGCTTGCAAACGGAGTCTAATC-3’), which anneals to the 3’ untranslated region (3’UTR).

Electrophoresis was applied to analyze RT-PCR products, using a 1.5% agarose gel that was stained with EZ-View stain. A 50 bp ladder (GeneDirex) was used to determine the size of RT-PCR products.

### Aphid transmission

**Fig. 3:**
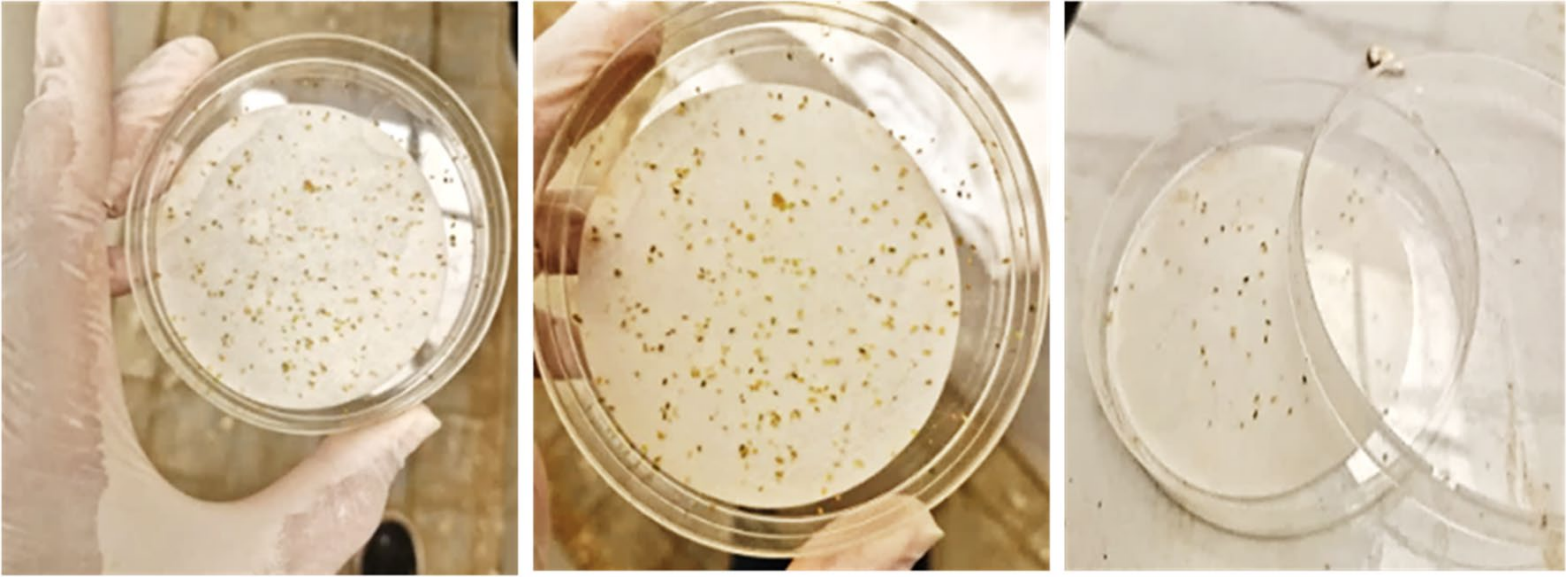
Aphids were starved for 2 h prior to acquisition access period (AAP).

Apterous aphids were starved for 2 h prior to virus acquisition (Fig. 3). They were then given an acquisition access period (AAP) of 2 to 3 min on ZYMV-infected source plants, followed by an inoculation access period (IAP) for 24 h. Ten aphids were used per test plant. After the IAP, the aphids were removed, and the test plants were sprayed with 0.01% malathion to eliminate any remaining insects. The plants were then maintained as a positive control under greenhouse conditions for symptom observation.

### Effect of RBPS on ZYMV transmission by *A. gossypii*

**Fig. 4:**
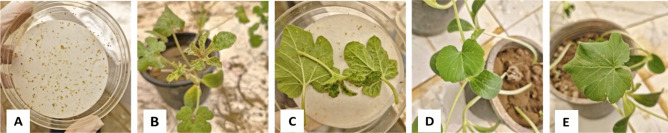
(**A**) Aphids starvation, (**B**) Virus source plants treated with RBPS, (**C**) Aphids AAP from treated virus source plants, (**D**) Transmission of viruliferous aphids to untreated healthy test plants, (**E**) Viruliferous aphids inoculate untreated healthy test plants (IAP).

**Fig. 5:**
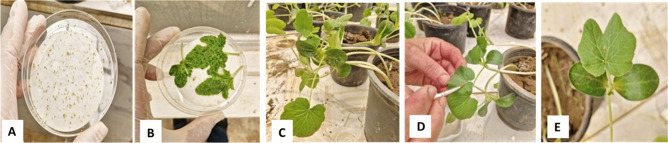
Effect of RBPS on ZYMV transmission by aphids (pre—inoculation treatment). (**A**) Aphids starvation, (**B**) Aphids AAP from untreated Virus source plants, (**C**) Healthy test plants treated with Rose Bengal, (**D**) Transmission of viruliferous aphids to treated healthy test plants, (**E**) Viruliferous aphids inoculate treated health test plants (IAP).

**Fig. 6:**
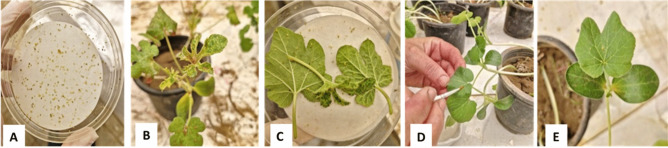
Effect of RBPS on ZYMV transmission (pre- acquisition and pre-inoculation treatment). (**A**) Aphids starvation, (**B**) Virus source plants treated with Rose Bengal, (**C**) Aphids AAP from treated virus source plants, (**D**) transmission of viruliferous aphids to treated healthy test plants, (**E**) Viruliferous aphids inoculate treated healthy test plants (IAP).

To evaluate the impact of the RBPS on zucchini yellow mosaic virus (ZYMV) transmission by aphids, three treatment strategies were tested:

1. Pre-acquisition treatment – RBPS was sprayed on the virus source plants before the aphids underwent the acquisition access period (AAP). The viruliferous aphids were, then, allowed an inoculation access period (IAP) on untreated healthy plants (Fig. 4).
2. Pre-inoculation treatment Aphids were allowed to acquire the virus from untreated virus source plants and then allowed an inoculation access period (IAP) on treated healthy test plants (Fig. 5).
3. Combined pre-acquisition and pre-inoculation treatment – RBPS was sprayed on both the virus source plants before the AAP and on the healthy plants before the IAP (Fig. 6).

In all treatments, groups of 10 aphids were used per test plant, and AAP of 2 to 3 min and IAP for 24 h were applied. After inoculation, aphids were removed, and the test plants were sprayed with 0.01% malathion to eliminate any remaining insects. All plants were maintained under greenhouse conditions and observed daily for up to 30 days to monitor symptom development and assess ZYMV transmission efficiency.

The transmission rate of virus was calculated using the following formula:

$$Rateoftransmission\% = \frac{{No.of\inf ected}}{{No.oftotaltestedplants}}$$

### The reduction in transmission

The reduction in transmission (%) was calculated by comparing the transmission rate of each treatment group to the positive control group according to the following formula:

$$\:{\mathop{\rm Re}\nolimits} duction{\mathop{\rm int}} ransmission = \left( {\frac{{Transmissionrateofpositivecontrol - Transmmisionrateoftreatmenyt}}{{Transmessionrateofpositivecontrol}}} \right) \times 100$$

### Statistical analysis

Duncan’s post hoc test was applied to calculate the mortality rates and evaluate significant differences for all treatments. The test was performed using SPSS computing program (Version 16, SPSS Inc., Chicago, IL, USA). The concentration-mortality response curve for probit analysis was conducted as described by Finney^41^. Bioassay data was pooled and analyzed (LC_50_ and LC_90_ confidence limit values) based on the methods described by Noack & Reichmuth^37,42^ The results are displayed as mean ± standard error with a 95% confidence level.

## Result and discussion

### Bioassay

The lethal effect of Rose Bengal photosensitizer (RBPS) on *A. gossypii* is indicated in (Table1). The mortality percentage increased with the increase of the concentration. The highest mortality observed, 100%, was recorded after 72 h of treatment with maximum concentration (1000 ppm). Rose Bengal is considered as an organic potassium salt that is the dipotassium salt of 2, 3, 4, 5 – tetrachloro-6—(2, 4, 5, 7—tetraiodo-6-hydroxy-3-oxoxanthen-9-yl) benzoic acid (Hopkin & Williams). It serves as a fluorochrome and a histological dye^43^.

**Table 1:** Corrected mortality percentage of *A. gossypii* adults treated with Rose Bengal photosensitizer (RBPS).

Photosensitizer (ppm)	Mortality % (Mean ± SE)	
	24h	72h
0.1	21.3 ± 0.75^a^	47.5 ± 1.19^a^
1	58.8 ± 0.75^b^	80.0 ± 0.41^b^
10	63.8 ± 0.48^c^	90.0 ± 0.82^c^
100	73.8 ± 0.48^d^	92.5 ± 0.29^d^
1000	90.0 ± 0.41^e^	100.0 ± 0.00^e^
F values	1853.571	906.69
P values	˂0.001	˂0.001

Means followed by letters represent significant differences between concentrations.

**Fig. 7:**
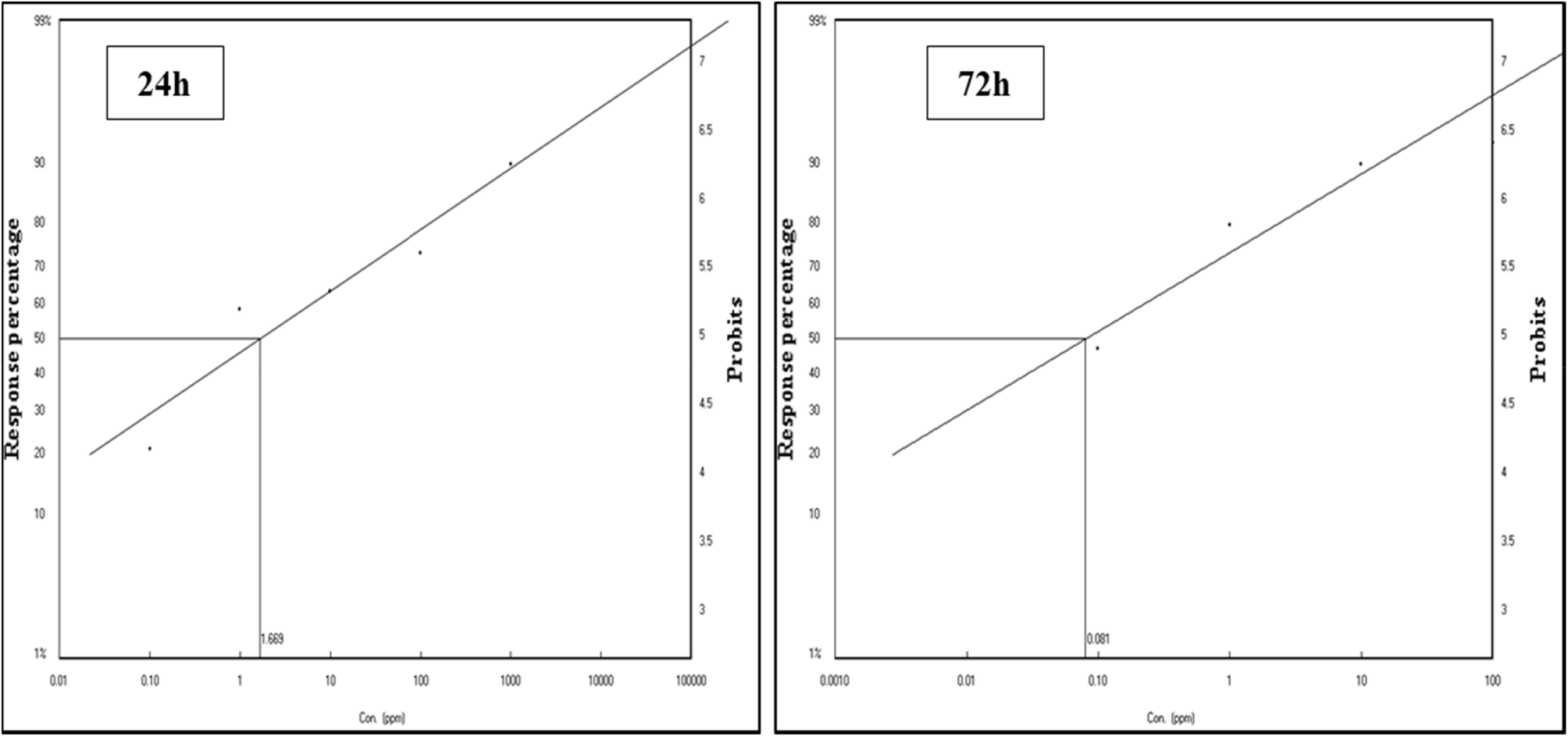
Lethal concentration of Rose Bengal photosensitizer (RBPS) for *A. gossypii* adults after 24 and 72 h under laboratory conditions.

Lethal concentration fifty of RBPS was 1.6688 and 0.0807 ppm after 24 h and 72 h, respectively, under sunlight exposure (Table 2) and (Fig. 7). This is in conformity with several research studies that have highlighted the toxic effect of RBPS on *Spodoptera littoralis* ^39^, *Culex pipiens*^44^ and *Hylemyia antiqua* ^(^^45^^)^. Also, Salem et al^43^. indicated that Rose Bengal (as a photosensitizer) mixed with soap can be used as a lethal agent against *Icerya aegyptiaca and Parlatoria ziziphus*. Moreover, El-Shourbagy et al^46^. confirmed the toxic effect of Rose Bengal photosensitizer on *A. gossypii* under laboratory conditions.

**Table 2:** Lethal concentration of Rose Bengal photosensitizer (RBPS) for *A. gossypii* adults after 24 and 72 h under laboratory conditions.

RBPS	LC _50_			LC _90_			Chi	Slope
	LC _50_	Lower limit	Upper limit	LC _90_	Lower limit	Upper limit		
24 h	1.6688	0.0545	9.2987	1215.0604	871.8144	1,656,061.5882	11.068	0.448
72 h	0.0807	0.0014	0.2088	13.6777	4.619	425.1119	7.826	0.575

### Mechanical inoculation and maintenance of virus isolate

**Fig. 8:**
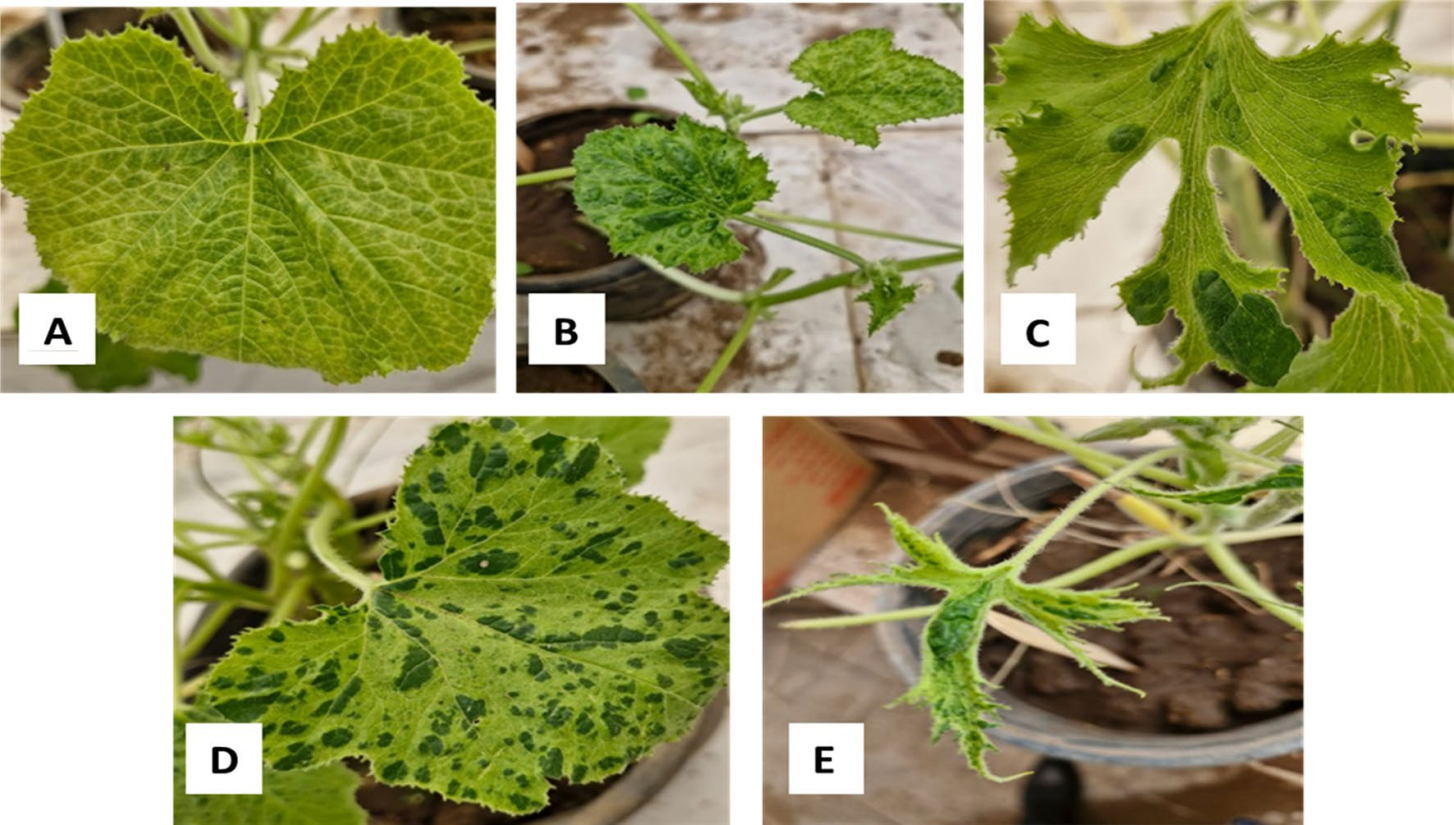
Symptoms on squash plants cv. Eskandrani after serial mechanical inoculation (**A**) vein clearing, (**B**) mosaic and blisters, (**C**) leaf deformation and blisters, (**D**) blistering, (**E**) shoestring leaf (filiform).

The results of the mechanical inoculation of squash plants *cv*. Eskandrani, using the isolate sample, involve vein clearing as a primary symptom, which disappeared. This was followed by mosaic, blistering, and leaf malformation which appeared 5–7 days after inoculation. Eventually, shoestring leaf symptoms appeared 8–14 days after inoculation (Fig. 8). These symptoms mimic those described on squash due to ZYMV infection worldwide, in France^47^, in the USA^48^, in New Zealand^27^, in Australia^23^, in India^49^, and in Egypt^50^. Non inoculated plants showed no virus symptoms.

### RT-PCR- based detection

**Fig. 9:**
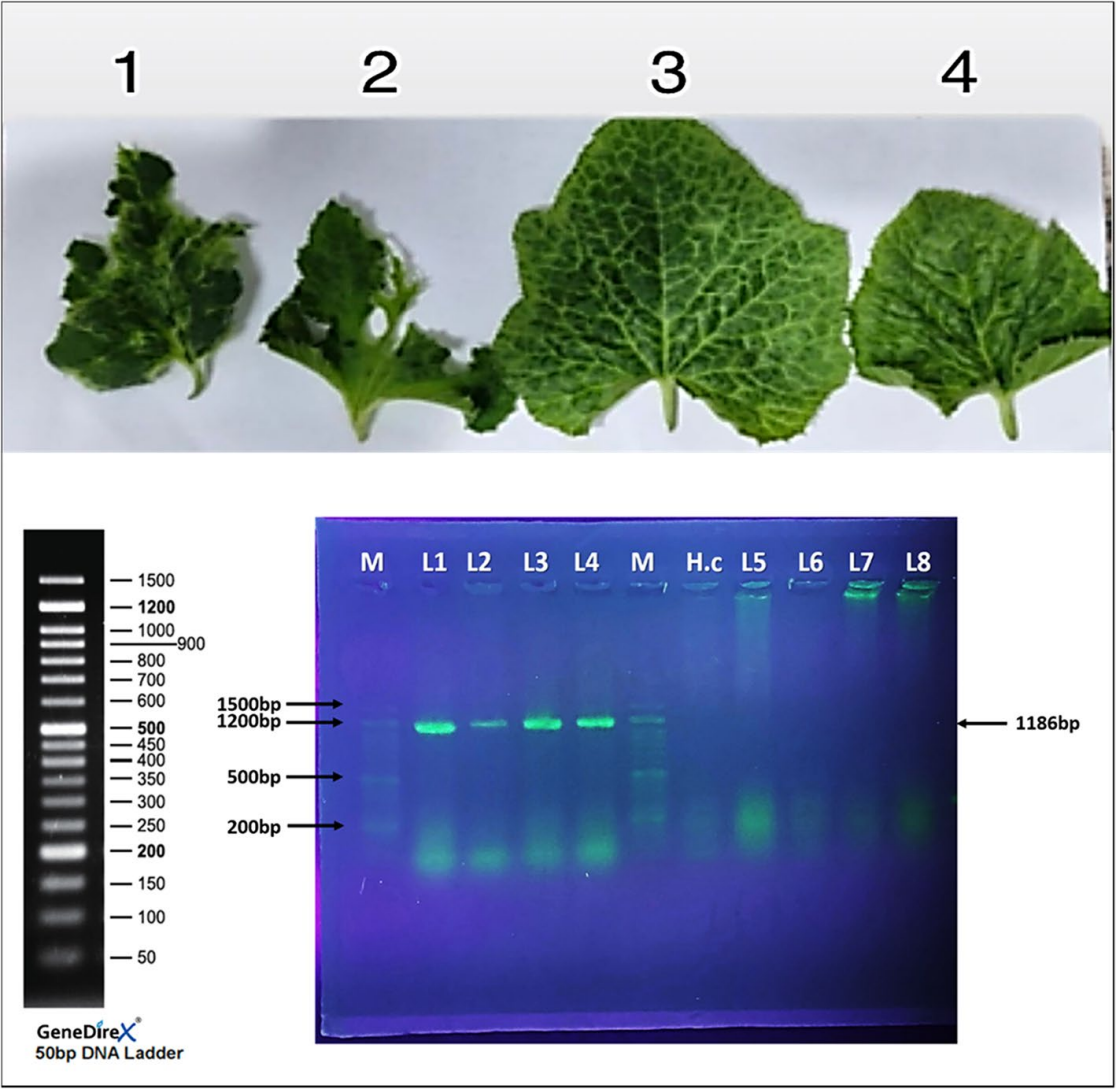
Agarose gel electrophoretic analysis of the RT- PCR products revealed the primers, ZYMV-specific primers (ZY-2 and ZY-3), amplified products of 1186 bp. (1–4) squash plants infected with ZYMV, (M) marker, (H.c) healthy squash plant. (5–8) non-viruliferous aphids.

**Fig. 10:**
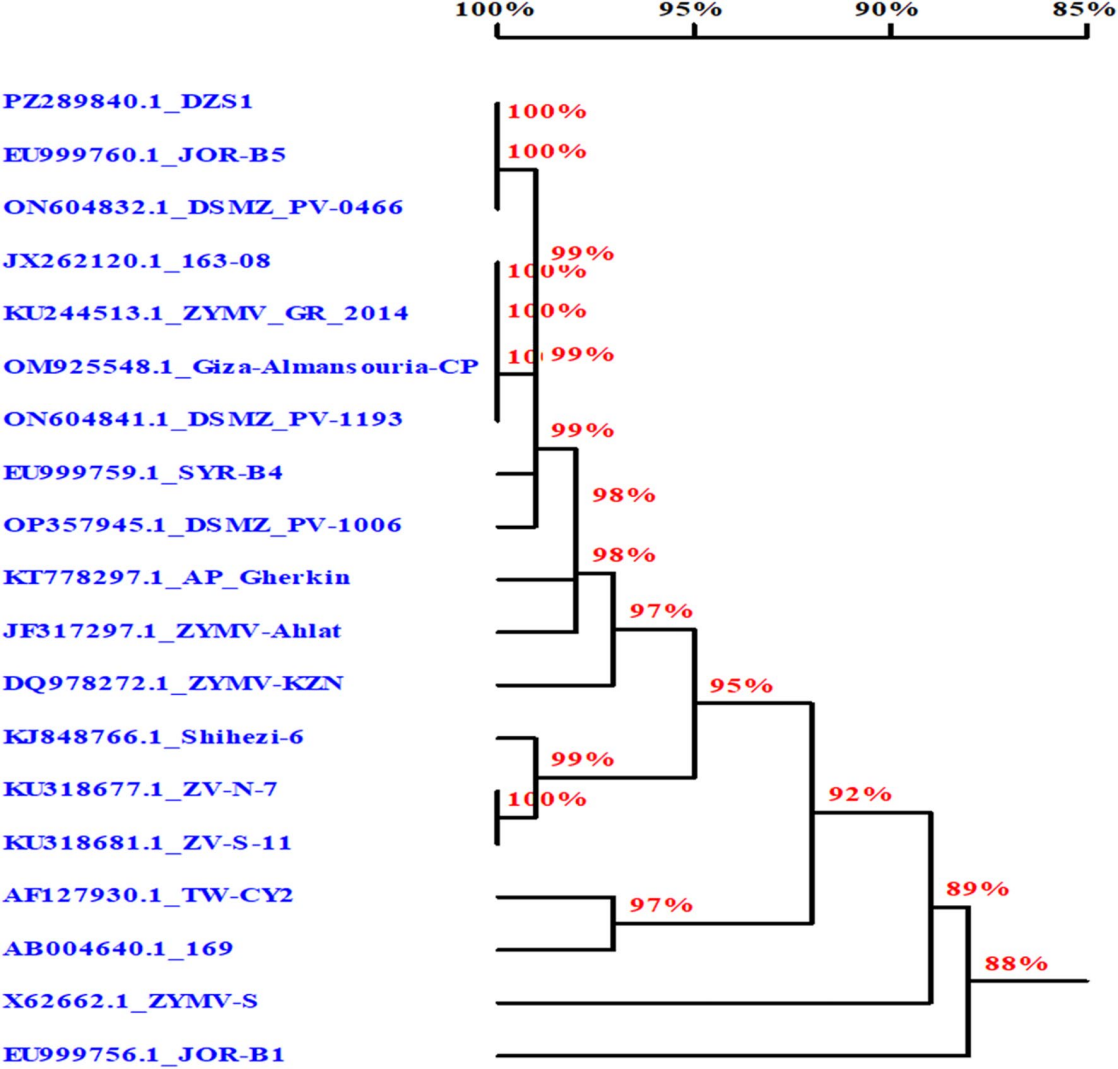
A phylogenetic tree demonstrating the evolutionary relationship between The Egyptian isolate DZS1 (PZ289840.1) and different available isolates from the GenBank database.

RT-PCR amplification was carried out using total viral RNA, which was extracted from four mechanically infected squash samples. Results revealed that ZYMV-specific primers ZY-2 and ZY-3, which were reportedly used for the detection of ZYMV^51–53^, amplified a specific fragment of approximately 1186 bp. This fragment contains the complete coat protein (CP) gene, part of the NIb gene, and most of the 3’UTR. No amplification was seen from healthy control (Fig. 9). ZYMV could be differentiated from other potyviruses infecting cucurbits, *i.e.,* watermelon mosaic virus type 2 (WMV-2), papaya ringspot virus type w (PRSV-W), and peanut mottle virus (PeMoV), by using the primer pair ZY-2 and ZY-3^51^. Sequence analysis was carried out using DNAMAN 7.0 software program (Lynnon BioSoft, Canada) and performing the phylogenetic tree (Fig. 10). This sequence was deposited in NCBI database with the accession number of PZ289840.1

## Effect of Rose Bengal photosensitizer (RBPS) on ZYMV transmission by *A. gossypii* Glov

### Pre-acquisition treatment

The data presented in Table 3 demonstrates that pre-acquisition treatment with RBPS reduced ZYMV transmission by *Aphis gossypii* compared to 93.3%, in the positive control (+). Whereas in pre-acquisition treatments, transmission rate was reduced to a range of 23.3% to 33.3%. This corresponds to a reduction in virus transmission ranging from 63.3% to 74.3%.

**Table 3:** Effect of pre- acquisition treatment of Rose Bengal photosensitizer (RBPS) on ZYMV transmission by *A. gossypii.*

Treatment	Replicate 1 (I/T)	Replicate 2 (I/T)	Replicate 3 (I/T)	Total (I/T)	Transmission rate (%)	Transmission reduction (%)
1	3/10	2/10	3/10	8/30	26.7	71.0
2	3/10	3/10	4/10	10/30	33.3	63.3
3	2/10	2/10	3/10	7/30	23.3	74.3
Negative control (–)	0/10	0/10	0/10	0/30	0.0	-
Positive control (+)	9/10	10/10	9/10	28/30	93.3	-
Mechanical inoculation	10/10	10/10	10/10	30/30	100.0	-

The Transmission Rate shows the percentage of plants that became infected after aphid exposure.

Reduction in Transmission compares each treatment’s transmission rate to the positive control, indicating how much the Rose Bengal treatment reduces virus acquisition by aphids.

These results suggest that the application of RBPS prior to the aphid AAP reduces virus acquisition. This observed reduction in ZYMV transmission rate could be attributed to the inhibitory effect of the RBPS on the aphids’ efficiency in acquiring the virus from infected plants.

### Pre-inoculation treatment

The results indicated in Table 4 show that pre-inoculation treatment with RBPS reduced the transmission rate of ZYMV by viruliferous *A. gossypii*. The transmission rates of the virus ranged from 40% to 43.3%**,** representing a transmission reduction of 51.9% to 55.6% compared to 90% in the positive control and 100% with mechanical inoculation. No ZYMV symptoms were observed in the negative control plants. These findings suggest that the application of RBPS before the IAP decreases the inoculation efficiency of ZYMV by viruliferous *A. gossypii*. This reduction may be due to the potential interference of the RBPS with the ability of the viruliferous aphids to effectively inoculate the virus into the plant.

**Table 4:** Effect of pre inoculation treatment of Rose Bengal photosensitizer (RBPS) on ZYMV transmission by *A. gossypii.*

Treatment type pre-IAP	Replicate 1 (I/T)	Replicate 2 (I/T)	Replicate 3 (I/T)	Total (I/T)	Transmission rate (%)	Transmission reduction (%)
1	6/10	4/10	3/10	13/30	43.3	51.9
2	5/10	4/10	4/10	13/30	43.3	51.9
3	6/10	3/10	3/10	12/30	40.0	55.6
Negative control (–)	0/10	0/10	0/10	0/30	0.0	—
Positive control (+)	8/10	10/10	9/10	27/30	90.0	—
Mechanical inoculation	10/10	10/10	10/10	30/30	100.0	—

The Transmission Rate shows the percentage of plants that became infected after aphid exposure.

Reduction in Transmission compares each treatment’s transmission rate to the positive control, indicating how much the Rose Bengal treatment reduces virus acquisition by aphids

### Combined pre-acquisition and pre-inoculation treatment

The effect of RBPS treatment, before both aphid acquisition and inoculation, on ZYMV transmission is summarized in (Table 5). Transmission rates in the treated groups ranged from 13.3% to 20.0%, representing up to an 86.7% reduction in transmission compared to the positive control. In contrast, both the positive control and mechanical inoculation resulted in 100% transmission, while the negative control showed no symptoms, confirming the absence of ZYMV in un-inoculated plants. These findings indicate that spraying RBPS prior to both the acquisition and inoculation access periods significantly reduced ZYMV transmission by *A. gossypii*.

**Table 5:** Effect of pre-acquisition and pre-inoculation treatment of Rose Bengal photosensitizer (RBPS) on ZYMV transmission by *A. gossypii.*

Treatment type	Replicate 1 (I/T)	Replicate 2 (I/T)	Replicate 3 (I/T)	Total (I/T)	Transmission rate (%)	Transmission reduction %
1	2/10	2/10	1/10	5/30	16.7	83.3
2	1/10	3/10	2/10	6/30	20.0	80.0
3	1/10	1/10	2/10	4/30	13.3	86.7
Negative control (–)	0/10	0/10	0/10	0/30	0.0	-
Positive control (+)	10/10	10/10	10/10	30/30	100.0	-
Mechanical inoculation	10/10	10/10	10/10	30/30	100.0	-

The Transmission Rate shows the percentage of plants that became infected after aphid exposure.

Reduction in Transmission compares each treatment’s transmission rate to the positive control, indicating how much the Rose Bengal treatment reduces virus acquisition by aphids.

Comparing the results of the combined treatment to the 2 other treatments, it is evident that the combined treatment is superior in reducing transmission rate. The pronounced decrease in virus transmission compared to the previous two treatments may result from the RBPS’ simultaneous impact on the aphids’ capacity to both acquire and inoculate the virus. This combined treatment appears to be the most effective strategy for reducing virus spread and as such we suggest that applying RBPS prior to both the acquisition and inoculation access periods is a recommended strategy for controlling ZYMV transmission by *A. gossypii*.

It is well known that plant viruses have, so far, no cure, therefore, effective management strategies continue to mainly rely on controlling their vectors and eliminating alternate hosts of viruses^29–31^. Reducing the population of vectors helps to limit virus transmission between plants. Many common insect pests have developed resistance to existing pesticides, making pest and disease management increasingly ineffective and costly, thus, it is imperative to explore and implement innovative alternatives. Photosensitizers, due to their unique mode of action, may offer an effective means of controlling insect pests with no associated risk of resistance development^54^. Several papers have focused on photosensitizers and their impact on insect pests including those that play a role in plant virus transmission. Ben Amor and Jori^55^ found that the photosensitizers, such as Rose Bengal and rhodamine B, which belong to the halogenated xanthenes, are effective photo-insecticides against at least two dozen insect species. Later, Pieterse et al.^54^ verified that the photosensitizer SUN-D-06 PS decreased the Western Flower Thrips (WFT) population to below threshold. In addition, El-Shennawy et al*.*^56^ showed the high activity of Rose Bengal, Rhodamine B, and Methylene Blue against the second instar larvae of *Earias insulana* under laboratory conditions. Recently, Gaber and El-Rahman^57^ assessed and confirmed the effectiveness of two photosensitizers, Magnesium Chlorophylline (Mg-Chl) and Copper Chlorophylline (Cu-Chl), against the adult aphid, *Rhopalosiphum padi*. Recently, Shehata et al.^58^, confirmed the efficacy of both photosensitizers, Rose Bengal and methylene blue as biocidal agents against *Anopheles pharoensis*at various developmental stages through strong binding affinity of them to acetylcholinesterase. Also Maharana et al.^59^, suggested that methylene blue trihydrate has a promising effect in controlling mosquito-borne disease vectors, thanks to its dual action of phototoxicity and inhibition of acetylcholinesterase 1 (AChE1), thus providing an environmentally friendly alternative to traditional insecticides.

The body of literature includes several studies on the photosensitizers and its potential role in environmentally sustainable pest management strategies. There is, however, very limited information on the application of photosensitizers to specifically interfere with insect-mediated plant virus transmission. Most control strategies of plant viruses focus either on vector suppression or host resistance. The integration of photodynamic principles into plant virus management, particularly targeting the transmission phase, remains largely unexplored. The present study contributes to this emerging research direction by, for the first time in our knowledge, evaluating the potential of Rose Bengal as a photosensitizer to reduce aphid-mediated transmission of ZYMV under controlled experimental conditions.

## Conclusion

Rose Bengal photosensitizer (RBPS) plays a vital and effective role in limiting the spread of the zucchini yellow mosaic virus (ZYMV) on zucchini plants. It is toxic and lethal to the cotton aphid, *A. gossypii*, the virus vector, and it also prevents transmission from infested to healthy plants. Therefore, this substance can be included in integrated pest management programs to prevent and control damage caused by aphid infestations and the spread of the ZYMV.

## Supplementary Information

Supplementary Information.

## Data Availability

The datasets generated and/or analysed during the current study are available in Sameha A. Metwally repository, accession number of PZ289840.1
