# Supplementary material for: Using Rose Bengal photosensitizer for controlling Aphis gossypii and reducing transmission of zucchini yellow mosaic virus on squash plants
Source: Sci Rep. 2026 May 19;16:15508. doi: 10.1038/s41598-026-51365-6 (PMC13187022; doi:10.1038/s41598-026-51365-6)
Supplement: Supplementary file 1 — Supplementary Information. [file 41598_2026_51365_MOESM1_ESM.docx]

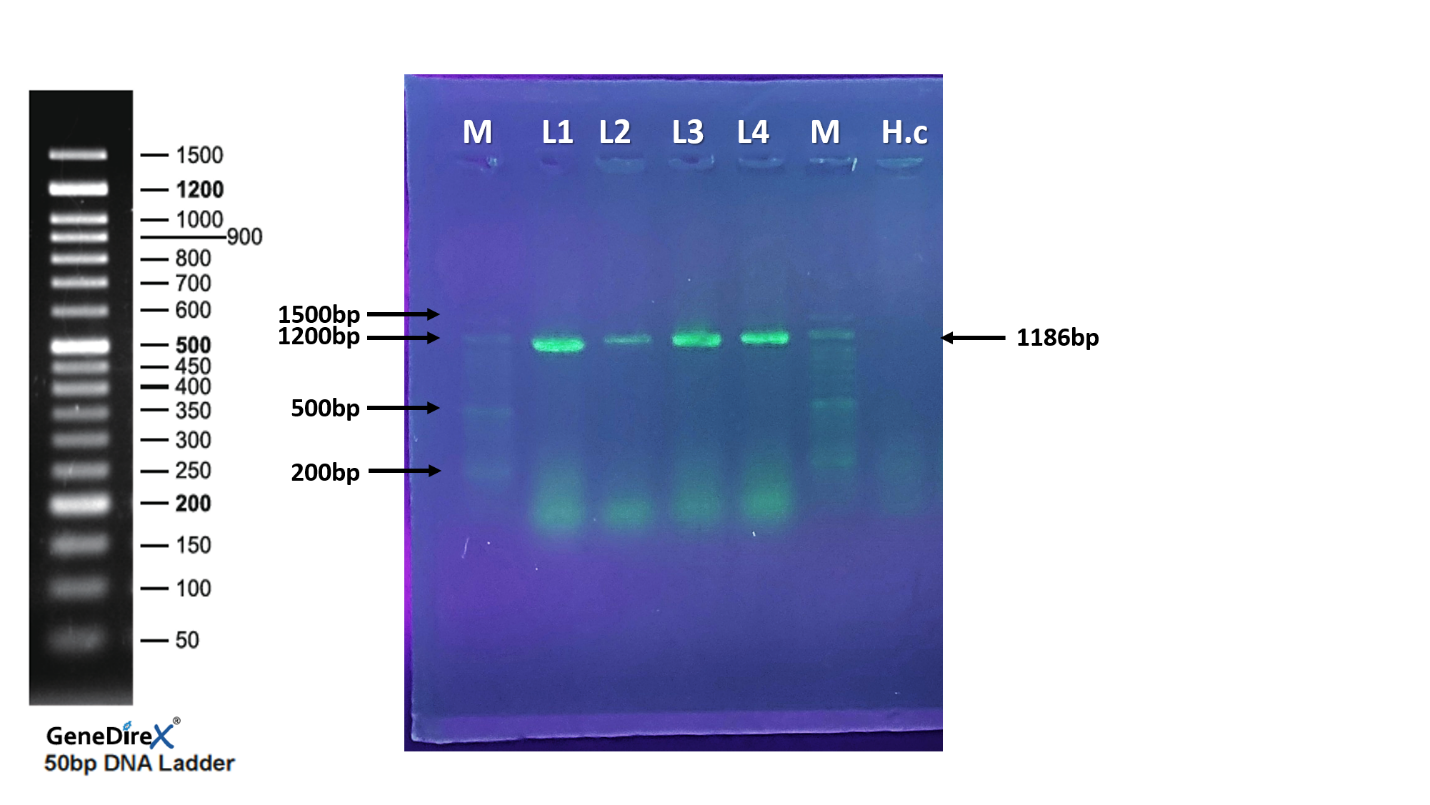


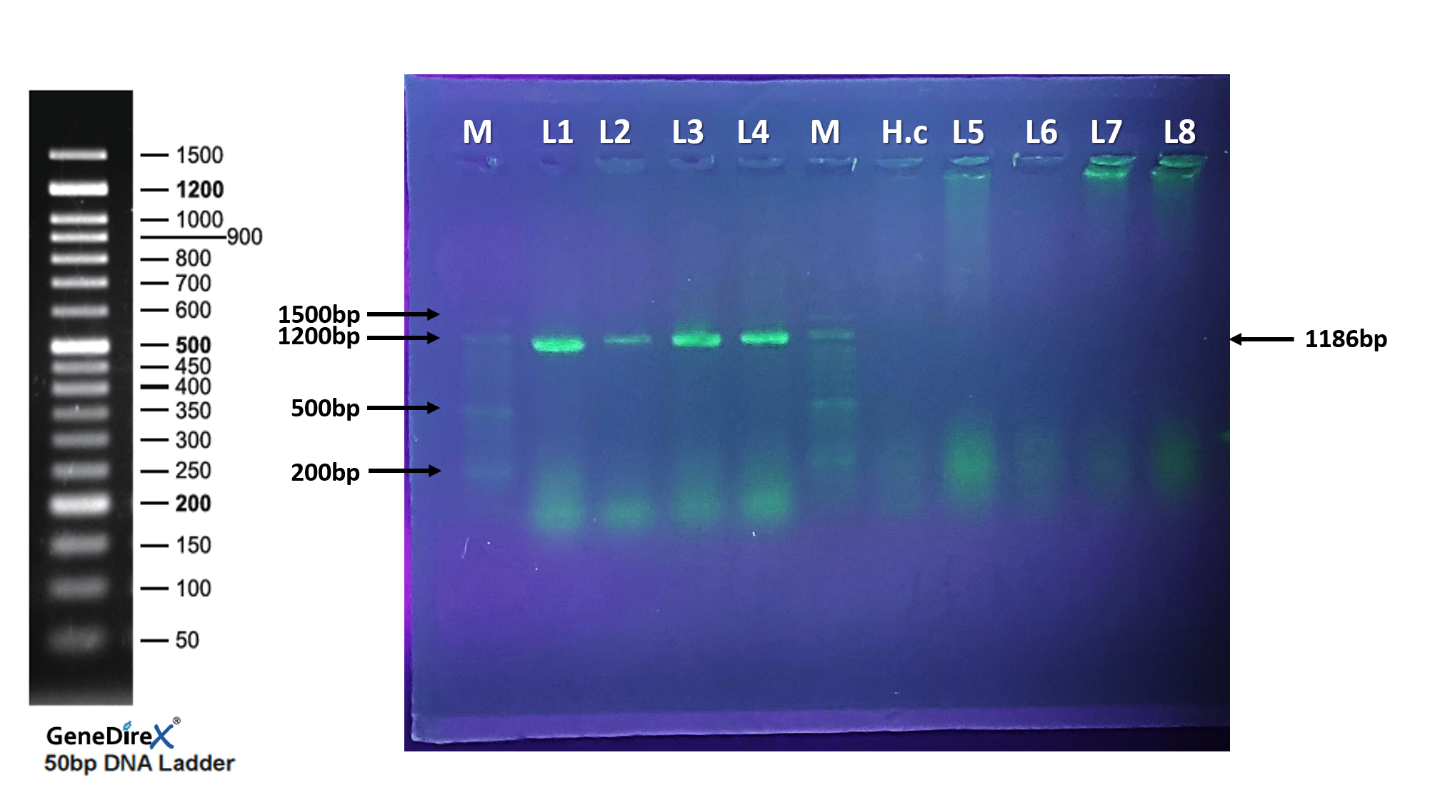


**Fig. (9): Agarose gel electrophoretic analysis of the RT- PCR products revealed the primers, ZYMV-specific primers (ZY-2 and ZY-3), amplified products of 1186 bp. (1-4) squash plants infected with ZYMV, (M) marker, (H.c) healthy squash plant. (5-8) non-viruliferous aphids.**
